# Observations on the Changing Shape of the Ice Mass and the Determination of the Sublimation End Point in Freeze-Drying: An Application for Through-Vial Impedance Spectroscopy (TVIS)

**DOI:** 10.3390/pharmaceutics13111835

**Published:** 2021-11-02

**Authors:** Bhaskar Pandya, Geoff Smith, Irina Ermolina, Evgeny Polygalov

**Affiliations:** DMU Lyo Group, School of Pharmacy, De Montfort University, Leicester LE1 9BH, UK; p09273780@my365.dmu.ac.uk (B.P.); IErmolina@dmu.ac.uk (I.E.)

**Keywords:** freeze-drying, primary drying endpoint, thermocouple, single-vial, process analytical technology

## Abstract

Models for ice sublimation from a freeze-drying vial rely on the assumption of a planar ice interface up to ~25% loss of ice mass (which is difficult to qualify) whereas single-vial determinations of the sublimation endpoint (by temperature sensors) are based on the point when the observed temperature reaches a plateau, which cannot differentiate between sublimation and desorption-drying. In this work, the real part capacitance of TVIS vial(s) containing frozen water (during sublimation drying) was measured at 100 kHz. This parameter $C^{\prime }\left(100\;\mathrm{kHz}\right)$ was shown to be highly sensitive to the shape and volume of the ice mass and is therefore a useful parameter for monitoring ice sublimation. By placing a digital camera in front of an isolated TVIS vial containing ice, it was possible to relate the changes in the shape of the ice mass with the changes in the trajectory of the time profile of $C^{\prime }\left(100\;\mathrm{kHz}\right)$ and determine the point of deviation from a planar ice interface and ultimately determine the point when the last vestiges of ice disappear. Thereafter, the same characteristics of the $C^{\prime }\left(100\;\mathrm{kHz}\right)$ time-profile were identified for those TVIS vials located out of sight of the camera in a separate full-shelf lyo study, thereby obviating the need for photographic examination.

## 1. Introduction

Freeze drying or lyophilization is a multi-stage down-stream processing method for preserving unstable liquid drug formulations (e.g., biopharmaceutical drug products such as monoclonal antibodies) [1]. The formulated drug solution is contained in glass containers (e.g., vials) which are placed on temperature controlled stainless steel shelves in a hexagonal array within a stainless-steel frame that prevents the edge rows of vials from falling off the edge of the shelf. The batch is then subjected to (i) freezing to form ice and crystallize out any solutes; (ii) primary drying to remove ice by sublimation; and (iii) secondary drying to remove the unfrozen water via diffusion and desorption [2].

Since the introduction of the US Food and Drugs Administration (FDA) Process Analytical Technology (PAT) initiative in 2003 [3] there has been much interest in the development of in-line measurement techniques for freeze-drying process development. Comprehensive reviews of batch and single vial technologies have been provided by Fissore et al. [4] and Nail et al. [5] A mathematical model [6] based on the classical heat and mass balance equation has also been developed to assist in freeze-drying process understanding and scale-up (Equation (1)).

(1) $$\frac{\Delta H_{s}A_{p}\left(P_{i}\left(T_{i}\right)-P_{c}\right)}{{\hat{R}}_{\mathrm{ps}}}=A_{v}K_{v}\left(T_{s}-T_{b}\right)$$

$\Delta H_{s}$ is the latent heat of ice sublimation, $A_{p}$ is the internal cross-sectional area of the vial (assumed to equal the area of the ice sublimation interface), $A_{v}$ is the is the horizontal external cross-sectional area of the vial through which heat is transferred from the shelf, $P_{i}$ is the vapor pressure of ice at the sublimation interface (which is a function of $T_{i}$, the temperature at the sublimation interface), $P_{c}\;$ is the chamber pressure (which is taken as a measurement of the 100% saturated vapor pressure of water in the drying chamber), ${\hat{R}}_{\mathrm{ps}}\;$ is the combined resistance of the dry layer resistance ($R_{p}$) and the stopper resistance ($R_{s}$), $K_{v}$ is the vial heat transfer coefficient, $T_{s}$ is the shelf surface temperature, and $T_{b}$ is the product temperature at the bottom centre of the vial [7]. Recent applications of this model account for key sources of freeze-drying heterogeneities, such as $T_{s}$, $K_{v},\;{\hat{R}}_{\mathrm{ps}}$, fill volume, the vial locations, and the bottom contours of the vial [7,8,9].

### 1.1. Assumptions in the Heat and Mass Transfer Model

Here we highlight some of the assumptions in application of the heat and mass balance equation for the determination of $K_{v}$ and $R_{p}$:

(1) The ice interface is expected to be planar over the initial sublimation period, and into the steady state period, and therefore the area term used in the determination of $R_{p}$ for the sublimation interface should be equal the internal cross-sectional area of the vial
(2) The vial is surrounded by its nearest neighbor in a hexagonal array and the relative contributions to $K_{v}$ from the various heat sources remain constant.
(3) The above conditions continue to apply up to the first 25–30% loss of ice mass.

It is important to recognize, whether it’s the determination of $K_{v}$ or $R_{p}$, that changes in the shape of the ice mass or drying rate (and therefore the steady state period) will vary across the shelves and between shelves, as a consequence of the contributions from side wall radiation and gas conductivity within the chamber. The result is that sublimation rates are generally higher, and the deviation from a planar sublimation interface is likely to occur sooner for the edge vials compared with the core vials [6]. The period corresponding to 25% ice sublimation should therefore be assessed for the edge vials. Then, when applying mathematical models to a batch of vials, one has to assume a multi-population model in which drying rates vary depending on the vial’s vicinity to either the front door or the metal guard rail or to the hotter vial not in contact with a rail, or whether the vial is part of the core vial population. For each cohort, models parameters (e.g., $K_{v}$, $R_{p}$) and a design space need to be established [10,11,12].

Drying rates of individual vials containing pure water/ice can be observed using a microbalance technique [13] and from the drying rate profile it can be inferred that the shape of the ice mass has changed. However, the technique involves the disruption of the packing of the vials, from the placement of the microbalance on the shelf of the dryer, in addition to an interruption to the process from a brief but repeated lifting of the vial from the primary heat source, i.e., the shelf [13]. Both factors could easily result in a change to the drying process and hence a reliance on this technique might be fundamentally flawed from the outset. 

Thermocouples could be used to define a period of temperature stability, from which a steady state may be inferred, but the additional heat contribution during drying and the impact on ice nucleation and growth during the freezing stage all contribute to difference in the way the TC-containing vials dry.

Neutron imaging and in situ tomography are able to scan the partially dried matrix in a glass vial and visualize the sublimation kinetics and in doing so provide true topographic information on the changing structure of the ice front. However, they require a line of sight to the vial(s) under scrutiny, which then impacts the drying process and results in anomalous ice shapes developing during the process [14].

It remains the case, that no technique has been established which can qualify the time period over which the sublimation interface remains unchanged, and steady state conditions apply, without perturbing the system that one is attempting to measure.

### 1.2. Primary Drying Endpoint

It is well known that primary drying is the longest phase of a lyophilization cycle and manufacturers should focus on optimizing primary drying to make the whole process more efficient [5,15]. In the remainder of this introduction, we focus on the methods used in the pharma industry for determining the primary drying endpoint in single vials. Most commonly this end point is estimated using thermocouples and resistance temperature detectors (RTDs) [5] and more recently, using wireless technologies such as the Temperature Remote Interrogation System (TEMPRIS^®^, Tempris GmbH, Holzkirchen, Germany) [4,16]. One might expect further adoption of these wireless technologies, now that robotic systems are available for loading the sensor into the vial, as an integral part of the filling line. On the fringe of these technologies, in terms of adoption/usage, are optical fiber sensors terminated by a Bragg grating (FBGs) [17].

All these technologies involve the insertion of a probe into the product to measure its ‘point’ temperature during the lyo cycle, with the sublimation end point being assessed after the product temperature increases above that of the shelf temperature, as the process of sublimative cooling ends [18]. However, there are several potential issues associated with the altered ice nucleation and faster drying kinetics of the thermocouple containing vials, which is exacerbated by the fact that the sensor containing vials are generally placed at the front of the vial stack for practical reasons, meaning that these vials are not representative of the vast majority of the batch [4,5,15]. Moreover, the end point is determined when the temperature reaches a plateau. However, given that the plateau extends over a period or a few hours then it becomes somewhat difficult to judge precisely when the end point has been reached.

The issues with these product-invasive probes has stimulated the development of non-product invasive, individual-vial sensors such as (i) plasma-sputtered thermocouples deposited externally to the glass vial [19]; (ii) Bragg-grating sensors embedded in the shelf [17]; (iii) soft sensor based on mathematical modelling [20]; (iv) heat flux monitoring using thermocouples externally attached to the base of an empty vial and a sample containing vial (drying process monitoring, DPM) [21] or to the top of the shelf and the vial bottom (heat flux transducer) [22]; (v) infra-red camera imaging system that employs a mechanistic model developed by Velardi et al. [23] to determine the product temperature distribution in a group of vials at the bottom of the product during primary drying, the primary drying endpoint, and certain process parameters [24].

Ultimately, any technique for the determination of sublimation end point which is based purely on changes in the thermal properties of the vial and contents will be compromised by the fact that the contribution to temperature changes from the desorption of water from the unfrozen fraction (leading to the attainment of thermodynamic equilibrium of water vapor distributed between the solid fraction and the gaseous phase) is conflated with that from the sublimation of ice. In other words, one process inevitably merges with the other.

Another non-invasive technique for the determination of primary drying end point is through-vial impedance spectroscopy (TVIS). In its current manifestation, a pair of thin film or metal foil electrodes are attached on opposite faces of the glass wall and connected to a purpose built broad-band (10 to 1 MHz) electrical impedance spectrometer [25]. The object under test is, in effect, a composite capacitor comprising the glass vial and its contents. When filled with water, the complex capacitance spectrum is characterized by a peak in the imaginary part capacitance (dielectric loss) and a step in the real part capacitance (dielectric storage) as a result of the interfacial (or Maxwell-Wagner) polarization of the glass wall through the electrical conductance of the water. For frozen water (ice), there is also a peak in the imaginary part and the step in the real part but in this case, it is the manifestation of the dielectric relaxation of the ice [25]. Previously, it has been demonstrated that TVIS can provide useful additional information on sublimation kinetics [26,27].

In this study, we present a universal parameter, $C^{\prime }\left(100\;\mathrm{kHz}\right)$, which is the real part capacitance measured at a frequency of 100 kHz, and describe opportunities for tracking, non-invasively, changes in the ice mass as drying progresses throughout the entire primary drying stage. To this end, we compared the time profile of $C^{\prime }\left(100\;\mathrm{kHz}\right)$ during the entire sublimation process with photographic evidence on a single vial and identified features that could be used to infer key stages in the evolving shape of the ice mass. These features include: (i) the period during which the area of the sublimation interface remained constant; (ii) the point when the ice mass no longer had contact with the inside wall of the vial (and only covered the base of the vial); (iii) the time point when the last vestiges of ice can be observed in the vial (i.e., the sublimation endpoint).

The rationale for choosing $C^{\prime }\left(100\;\mathrm{kHz}\right)$ is that the permittivity of ice on the high frequency side of the loss peak (which is known as the instantaneous relative permittivity, $\varepsilon_{\infty }$, of ice) has almost zero temperature dependence [28]. Given that the dielectric relaxation of ice spans approximately three decades in frequency, it follows that a measurement of the real part capacitance at a frequency which is of the order of 1–2 decades higher than the peak frequency would be close to providing an estimate for the instantaneous relative permittivity, $\varepsilon_{\infty }$. This has led us to the conclusion that we can effectively tune the TVIS (i.e., select a specific frequency) to provide a parameter which is purely dependent on ice mass as opposed to both ice mass and temperature (the peak frequency lies in the region 316 to 4 kHz for temperatures of −50 to −15 °C). Other frequencies, e.g., 200 kHz or higher, might also be used; provided suitable instrumentation is available for the precise measurement of the rather small electrical capacitance of what is in effect an empty glass vial. For example, a standard glass tubing vial of nominal volume 10 mL containing 3 g of frozen water, with electrodes of approximate dimension 10 × 19 mm (height × width) has a capacitance at high frequency that decreases from ~100 fF (prior to sublimation) to ~50 fF (at the end of sublimation). 

In future articles we shall demonstrate that the TVIS derived primary drying endpoint can be clearly differentiated from thermal changes associated with water loss from the unfrozen fraction and hence provide a precise assessment of the true sublimation endpoint in the freeze-drying of solutions.

### 1.3. Study Aim and Objectives

The aim of this work is to develop an application for through vial spectroscopy (TVIS) for monitoring the changes in the shape of the ice mass during the process of sublimation and for determining the endpoint of ice sublimation in TVIS vials at different locations of the shelf of a small-scale development dryer.

## 2. Materials and Methods

### 2.1. Freeze Drying of an Isolated TVIS Vial

**Figure 1:**
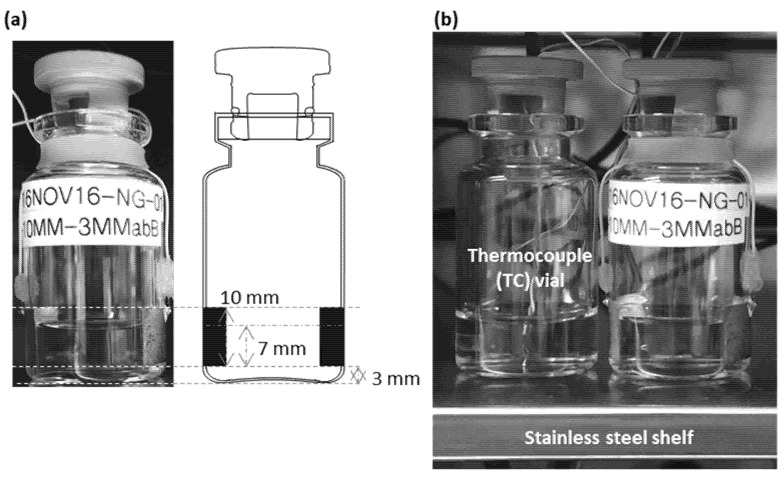
(**a**) TVIS vial containing 3 g of double distilled water, with copper electrodes (10 mm by 19 mm) positioned at 3 mm from the vial base; (**b**) TVIS vial and a type T thermocouple containing vial, each filled with 3 g of double-distilled water and placed at the front of the shelf of a Virtis Advantage Plus lab-scale dryer.

For the TVIS measurement, a Schott 10R Type 1 glass tubing vial (VC010-20C; Adelphi Ltd, Haywards Heath, UK) was modified with foil electrodes of dimensions 10 mm (height) by 19 mm (width) cut from a copper adhesive tape (3M 1181 Tape Copper Foil with Conductive Adhesive) and attached to the surface of the glass wall, at a distance of 3 mm from the bottom of the vial (Figure 1a). This TVIS vial and another unmodified vial containing a Type T thermocouple, were each filled with 3 g of double-distilled water and then placed at the front of the middle shelf of a Virtis Advantage Plus freeze-drier (Figure 1b). The TVIS vial was then connected to one of the channels on a five-channel impedance analyzer that has been designed with the specific requirements of a through vial impedance measurement [26]. The 3 g volumes of water were then freeze-dried using the lyo-cycle shown in Table 1, until all the ice had disappeared from both vials. Each 2 min throughout the entire freeze-drying cycle the following data were recorded:

(i) The complex capacitance spectra of the TVIS vial between 10 Hz and 1 MHz (with 10 data points per decade),
(ii) The freeze-dryer process parameters of the shelf temperature and chamber pressure and the product temperature from the thermocouple in the unmodified glass vial
(iii) Photographic images of the TVIS vial using a Canon DSLR camera

### 2.2. Freeze-Drying of a Batch of Vials including 3 TVIS Vials at Three Positions across the Shelf

**Figure 2:**
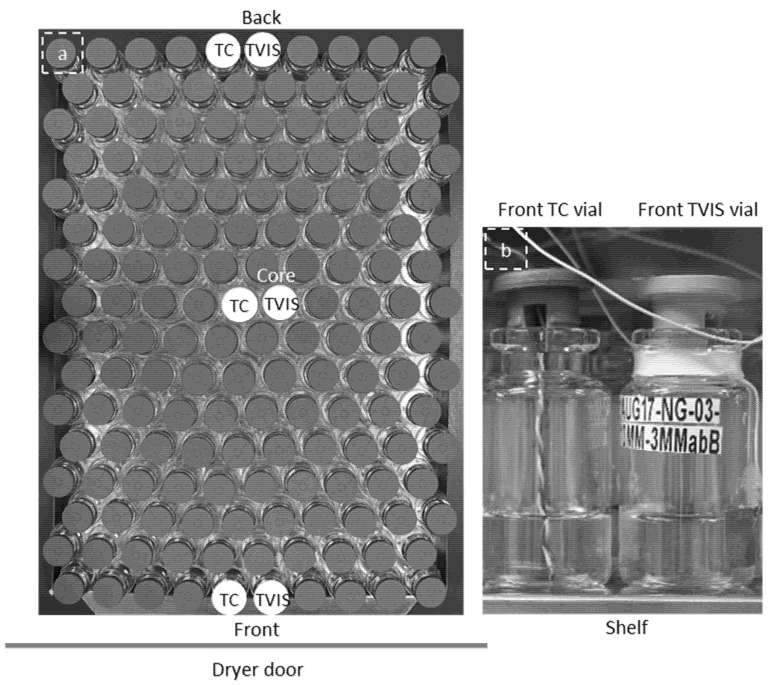
Freeze-drying of double distilled water in a batch of 160 × 10R vials: (**a**) Top view of the vial array showing the position of the TC and TVIS measurement vials; (**b**) the front TVIS vial and the nearest neighbor vial containing a Type T thermocouple with its sensing element in contact with the bottom center of the vial base.

A batch of 160 × 10R vials were filled each with 3 g of double distilled water. Of the 160 vials, three were TVIS vials with copper electrodes (19 mm by 10 mm) positioned at 3 mm from the base of the vial and hence from the freeze-drying shelf were placed in the front row (edge), the ninth row (i.e., core region) and the last row (edge) of the batch as shown in Figure 2a. Hereafter these vials are referred to as the front TVIS vial, the core TVIS vial, and the back TVIS vial. Each TVIS vial was accompanied by a nearest neighbor vial containing a Type-T thermocouple with the sensing element positioned in contact with the bottom-center of the vial base. A photograph of the front TVIS vial next to the nearest neighbor TC vial is shown in Figure 2b. Freeze drying was carried out in a Virtis Advantage Plus freeze dryer equipped with a five-channel TVIS system. The stages of the lyo cycle are given in Table 2. 

It should be pointed out that there are differences between the two lyo cycles. In the lyo cycle for the single vial, the shelf temperature is lowered to −40 °C and primary drying is carried out at −15 °C, whereas for the batch the shelf temperature is lowered to −45 °C and primary drying is carried out at −5 °C. The lower shelf temperature for the single/isolated vial ensures a slower drying rate and one that we hope might be comparable to the drying rate for the front vial in the batch experiment hence facilitating a more direct comparison of the drying profiles recorded by the TVIS system. 

## 3. Results and Discussion

### 3.1. Features of the Real Part Spectra during the Primary Drying Stage

**Figure 3:**
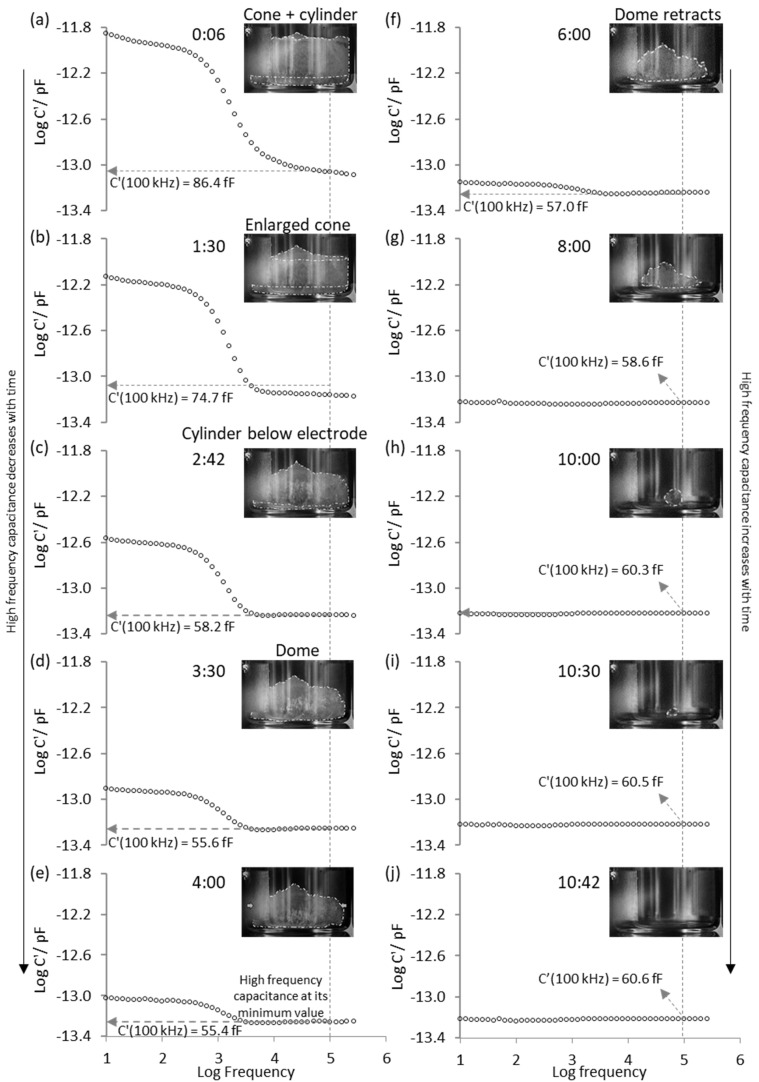
Bode plots (**a**–**j**) showing the real part spectra of ice contained in the TVIS vial with electrodes 10 × 19 mm (at 3 mm from the vial base) and the photographs of the sublimation front obtained at various time-points during primary drying. Features of the time profile of the $C^{\prime }\left(100\;\mathrm{kHz}\right)$ parameter during primary drying stage.

Figure 3 shows example spectra of the real part capacitance for the single (isolated) TVIS vial containing frozen water (ice) obtained at various time-points over the first 10–11 h of primary drying, alongside photographs of the shape/characteristics of the ice mass at the corresponding time points. Three observations can be made with respect to the progression of the sublimation process. 

The first is that the magnitude of the dielectric relaxation process (i.e., the height of the step in the real-part capacitance) decreases during primary drying. This might be expected given that the process is due to the dielectric relaxation of ice and so as the ice volume decreases within the electrode region then one would expect to see a progressive decrease in the magnitude of this process. 

The second observation is that the relaxation process decreases to zero before all the ice has been removed from the vial. In other words, the absence of a measurable relaxation does not in itself indicate the sublimation end point. 

The third, and possibly unexpected observation, is that the magnitude of the real part capacitance ($C^{\prime }\left(100\;\mathrm{kHz}\right)$) measured on the high frequency side of the relaxation process (in our case at a frequency of 100 kHz or a log frequency of 5) decreases from 86.4 fF at 0:06 h (6 min) to a minimum of 55.4 fF at 4:00 h and then increases to 60.6 fF at 10:42 h, and thereafter stays constant. 

The photograph alongside each spectrum suggests that:

- The minimum in $C^{\prime }\left(100\;\mathrm{kHz}\right)$ corresponds to the point when there is no more contact between the ice and the side wall of the vial;
- The unexpected recovery of the capacitance might be associated with the retracting of the ice mass towards the center of the vial base;
- The point when $C^{\prime }\left(100\;\mathrm{kHz}\right)$ remains constant corresponds to 100% ice sublimation.

A closer examination of these features is undertaken in the next section, with the time profile of $C^{\prime }\left(100\;\mathrm{kHz}\right)$.

**Figure 4:**
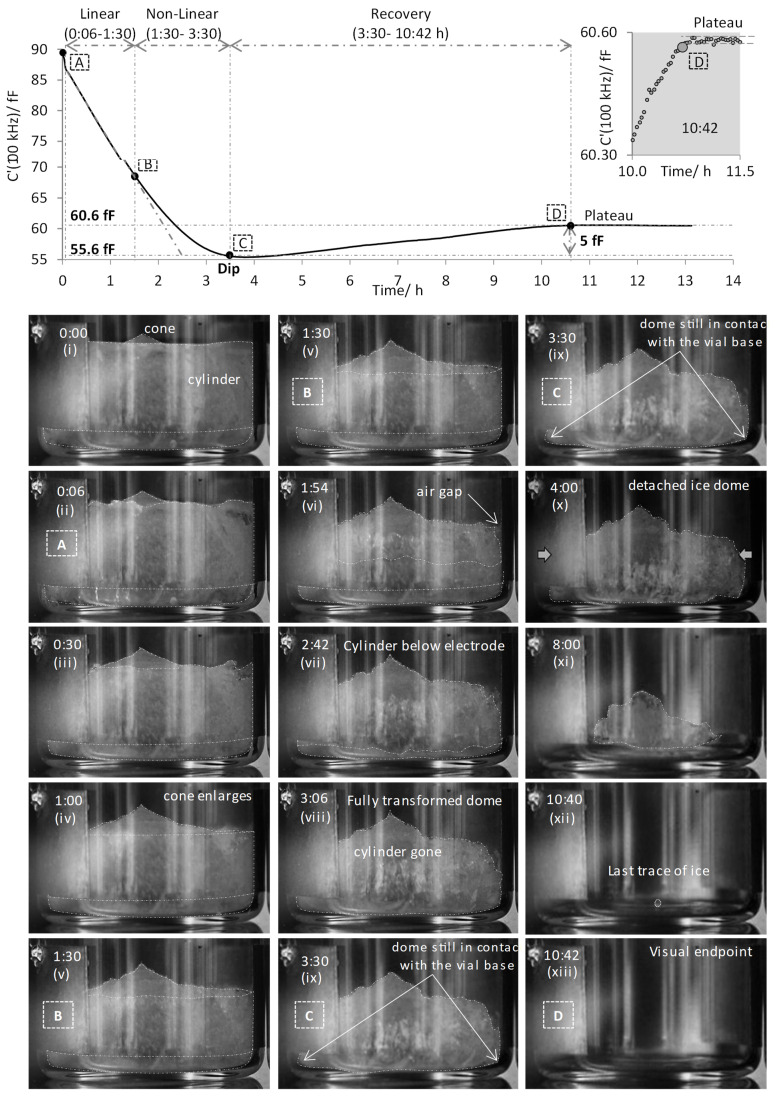
Mapping the facets of the $C^{\prime }\left(100\;\mathrm{kHz}\right)$ time-line for ice contained in the TVIS vial (with electrodes attached externally to the glass wall at a distance of 3 mm from the vial base) on the photographs of the ice sublimation interface during the primary drying phase: The graph at the top shows the time-line of $C^{\prime }\left(100\;\mathrm{kHz}\right)$ with the phases marked A to B, B to C and C to D corresponding to the linear phase, the non-linear phase and the recovery phase, respectively. Shown below are the photographs (**i**–**xiii**) of the sublimation front at various time-points. The plateau at point D corresponds to the visual endpoint of ice sublimation (10:42).

Figure 4 show the timelines of the $C^{\prime }\left(100\;\mathrm{kHz}\right)$ parameter during the primary drying stage for the single (isolated) TVIS vial containing 3 g of ice at the beginning of primary drying. A composite of the photographs of the sublimation front taken at various time-points during primary drying is presented below the timeline of $C^{\prime }\left(100\;\mathrm{kHz}\right)$ in order to map the changes in the shape of the sublimation front onto the different facets of the $C^{\prime }\left(100\;\mathrm{kHz}\right)$ timeline. The key stages and the corresponding photographs have been marked by letters enclosed within boxes. It is clear from Figure 4 that the magnitude of $C^{\prime }\left(100\;\mathrm{kHz}\right)$ decreases linearly over the first ~1.5 h min of primary drying, i.e., from point A (0:06) to point B (1:30), referred to as the linear phase. The photographs (i to v) taken between 0:00 and 1:30 demonstrate that the ice mass at first appears as a cylinder in intimate contact with the glass wall, with a small cone on the top surface which provides the initial sublimation interface. During this period the sublimation interface recedes down the vial but remains largely unchanged in terms of its shape and hence surface area. This might be expected on condition that the relative heat contributions from the various heat sources do not change [6].

However, from point B (1:30) to C (3:30), the trajectory of $C^{\prime }\left(100\;\mathrm{kHz}\right)$ becomes non-linear as the rate of change in $C^{\prime }\left(100\;\mathrm{kHz}\right)$ decreases. Over this non-linear phase, some dramatic changes in the shape of the sublimation interface occur as demonstrated in the photographs v to ix: The preferential removal of the ice mass from the side wall results in the loss of the glass wall-ice contact as well as the ice cylinder decreasing in height, which is presumed to result from a greater contribution from heat radiation from the chamber walls and door [6] and from gas conduction in the partial vacuum surrounding the vial [8]. It is clear from photograph ix (at 3:30 h) that there is no longer an ice cylinder in contact with the side walls and what is left at that point is an ice dome that covers the entire base of the vial (i.e., the ice mass is still in contact with the points where the side wall meets the vial base, but it has no contact with the wall itself). This is also the point C when the drying curve of $C^{\prime }\left(100\;\mathrm{kHz}\right)$ reaches a minimum (55.6 fF), hereafter referred to as the “dip” in the timeline of $C^{\prime }\left(100\;\mathrm{kHz}\right)$.

**Figure 5:**
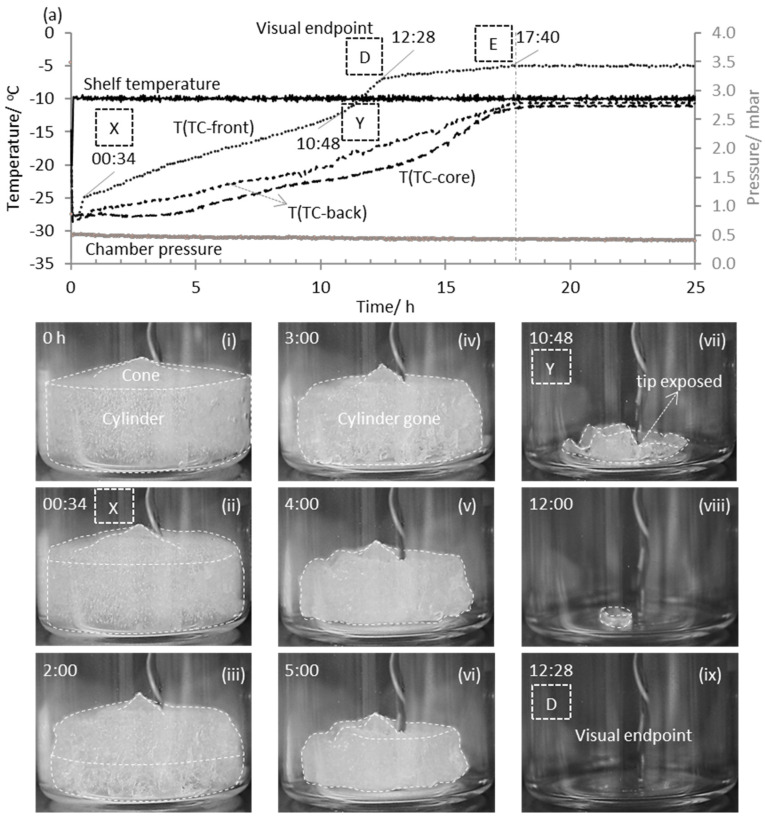
Determination of visual endpoint in the front edge vial containing a thermocouple and using this information to estimate the batch endpoint: (**a**) time-lines of the process parameters, namely the shelf temperature, the chamber pressure and the product temperature, T(TC-front), T(TC-back) and T(TC-core), at the bottom-center of the vial adjacent to each of the three TVIS vials located at the front edge of the shelf, the back edge of the shelf and in the core. Shown below this are photographs (**i**–**ix**) of the ice mass in the front TC vial taken at various time-points during primary drying. The labels X and Y enclosed by the dashed lines correspond to the inflections that indicate the detachment of the sensor from the ice layer in the immediate vicinity. The inflection at D and E correspond to the visual endpoint and the point at which all measurement vials reach equilibrium respectively.

During the period between point C (3:30) and point D (10:42), the capacitance profile changes course as the magnitude of $C^{\prime }\left(100\;\mathrm{kHz}\right)$ increases by 5 fF (i.e., from 55.6 fF to 60.6 fF) hereafter referred to as the recovery phase. The photographs ix to xiii show how the ice dome shrinks towards the center of the vial base as the primary drying progresses. It is interesting to note that $C^{\prime }\left(100\;\mathrm{kHz}\right)$ continues to recover until the last trace of ice is left in the vial (picture xii at 10:40). The recovery is completed when $C^{\prime }\left(100\;\mathrm{kHz}\right)$ reaches a plateau at point D (10:42) and corresponds to the visual endpoint of ice sublimation (as seen in photograph xiii). Closer examination of the end point is shown in the insert graph with the grey background (Figure 5). This enlargement of a selected portion of the graph shows that transition at the end point is quite distinct, with a sharp inflection from a steep, almost linear increase in $C^{\prime }\left(100\;\mathrm{kHz}\right)$ to a flat (time invariant) response. 

### 3.2. Observations of the Thermocouple and TVIS $C^{\prime }\left(100kHz\right)$ Profiles for a Batch of Vials

In the previous sections, we considered a single (isolated) TVIS vial to demonstrate the features of the high frequency capacitance timeline from the beginning of primary drying to the visual endpoint of this individual vial. In this section we consider a shelf full of vials (Figure 2) which includes 3 TVIS vials and 3 TC-containing vials, located at the front edge of the shelf, the core, and the back of the shelf. Here, we retain the nomenclature for labeling the transition points A–D from the isolated vial study described in the previous section but focus primarily the transition point D to mark the end point of ice sublimation. However, as will be described next, there was a necessity to mark another characteristic feature after the sublimation end point that we have assigned the letter E. Additional features that are unique to the TC profile will be labeled with the letters X and Y.

Let us first consider the response from the TC-containing vials. Figure 5 shows the time dependencies of the temperature sensed by the thermocouple in each of the three nearest neighbor vials, to the TVIS vials, alongside the shelf temperature and the chamber pressure. Also shown are the photographs of the TC containing front edge vial, obtained at representative time points throughout primary drying. The pictures for the front edge vial support the following interpretation of the TC temperature-time profile for that vial:

(i) The gradual detachment of the TC sensor from the ice mass, which might start as early as point X, which is followed with a notable inflection in the temperature profile at point Y, which appears to be associated with the full detachment of the sensing bead from the ice mass (Figure 5vii).
(ii) A clear inflection at point D which corresponds to the visual endpoint (Figure 5ix)
(iii) The visual end point is followed by gradual increase in temperature until it reaches a plateau at point E, which means that the vial has come to a thermal equilibrium with its surroundings (i.e., the shelf, the vial-array and the gas within the chamber).

The profiles for the back and the core vials share some of the features of the front vial, in that they appear to reach thermal equilibrium at point E at the same time as the front vial, however, the corresponding point D is less clearly defined.

**Figure 6:**
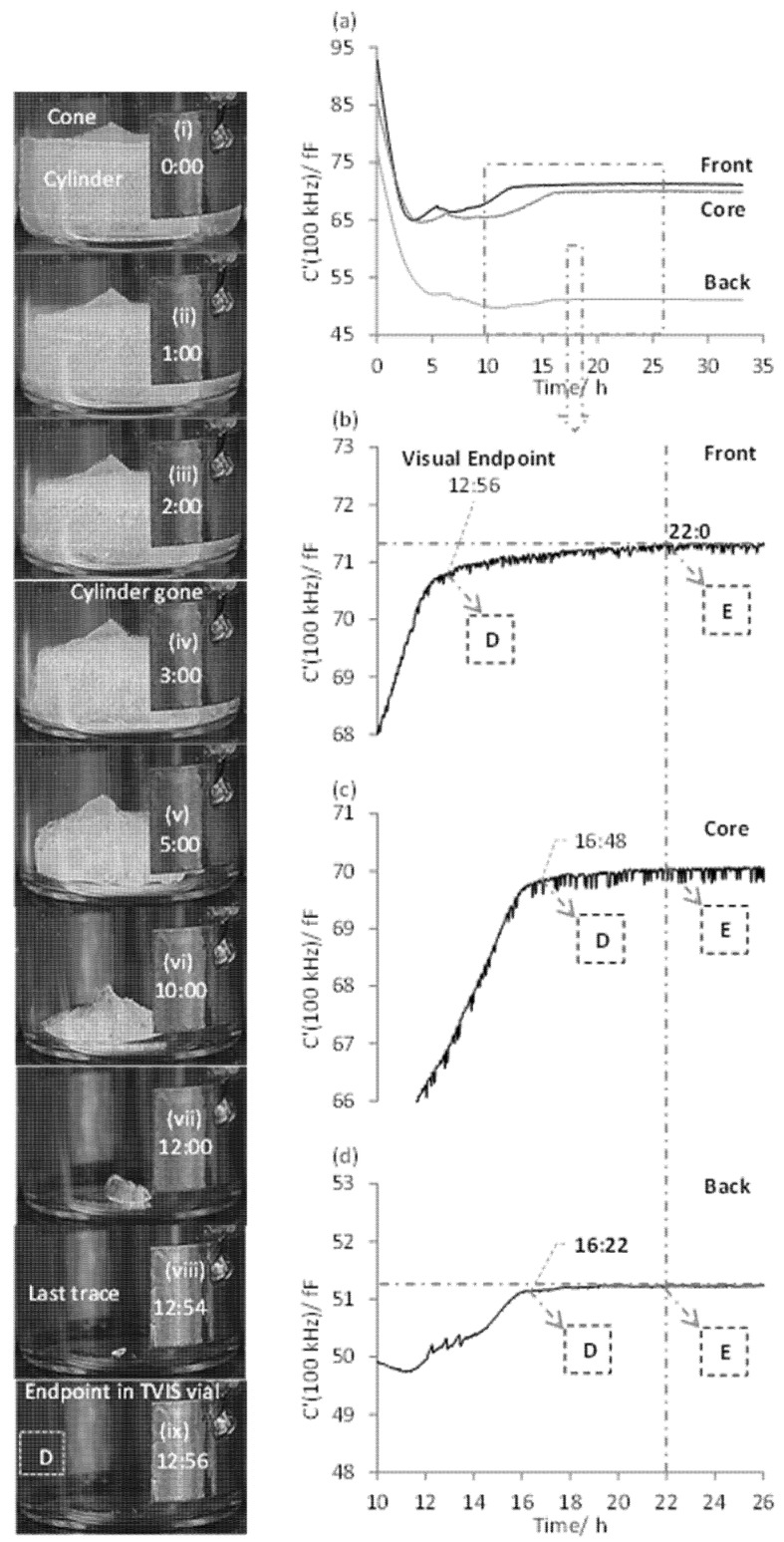
Determination of visual endpoint in the front edge TVIS vial and using this information to estimate the primary drying endpoint for the internal TVIS vials and therefore the batch endpoint: (**a**) an overlay of the time dependencies of $C^{\prime }\left(100\;\mathrm{kHz}\right)$ for the front, core and back TVIS vials; (**b**–**d**) time-lines of $C^{\prime }\left(100\;\mathrm{kHz}\right)$ for each TVIS vial on an enlarged scale. To the left are the photographs (**i**–**ix**) of the sublimation front obtained at various time-points during primary drying. The first inflection in the trajectory for the front TVIS vial corresponds to the visual endpoint (photograph ix at 12:56) and the second inflection at 22:00 occurs when all the TVIS vials reach equilibrium.

Next, we shall consider the timelines of the $C^{\prime }\left(100\;\mathrm{kHz}\right)$ parameter for the front, core and back TVIS vials, alongside the photographs of the sublimation front in the front edge TVIS vial (Figure 6). Plot (a) is an overlay of all three timelines for the front, core and back TVIS vials (over the entire primary drying stage: 0 to 35 h) whereas plots b, c and d correspond to the timeline close to the end point for each TVIS vial (i.e., between 10 and 26 h into the primary drying stage). The time profiles of all three vials are similar in some sense. In each case there is a steep rise in the high frequency capacitance to a transition point D, after which there is a more gradual increase in the high frequency capacitance, before reaching a plateau at point E. The point E occurs at approximately the same time for all vials irrespective of the position in the dryer. 

Photographic evidence from the front TVIS vial (photograph ix) qualifies that the end of the steep rise in the high frequency capacitance at 12:56 (point D in Figure 6b) corresponds to the visual endpoint. The subsequent, more gradual rise in the high frequency capacitance extends over a time period of ~8–9 h (and finishes at point E) during which time the others vials in the array are expected to reach their own sublimation end points. We can therefore expect the front vial to remain chilled to some extent during this period, even though it no longer contains ice and therefore there is no self-cooling of the vial from sublimation. The vials in the rest of the stack will be absorbing heat from their surroundings, including the front vial, and so the temperature of the front vial is expected to gradually increase as the population of the other vials come to the end of the sublimation process. 

Given that the electrodes are on the outside of the vial then the measured capacitance is inevitably influenced by the dielectric properties of the glass wall. It is the temperature dependency of the dielectric permittivity of the glass, at the excitation frequency of 100 kHz, that is being detected in the period from the end of ice sublimation within the TVIS vial in question (the front vial in this case) to the thermal equilibration point E. The point E therefore corresponds to the thermal equilibration of the vial with its surroundings. This may also be taken as the sublimation end point of the batch, but only for the specific case of the sublimation from pure water. 

For the TVIS vials located at the back and core of the array, it is then assumed that the sublimation end point may be also taken from the point D, and occurs at times 16:22 and 16:48, respectively. Although there is no direct photographic evidence to support this assumption, the fact that the point D for the core vial takes place 20 min after the back vial is consistent with the relative locations across the shelf of the dryer. 

As was the case for the TC containing vials, the thermal equilibration point E is also quite similar for all three TVIS vials, again pointing to the significance of this event as the time when the temperature becomes quite uniform and constant across the vial array. In general, it can be concluded that the edge vials do not reach thermal equilibrium until the internal vials reach their own equilibrium temperature. In other words, after the visual endpoint is reached, the temperature sensed by the front TVIS vial continues to stay low, as long as the center vials are still drying. 

## 4. Conclusions

In this study, a unique time profile of the real part capacitance of the TVIS vial, at an excitation frequency of 100 kHz, has been identified which displays characteristics that reflect key features of the evolving shape of the ice mass during the primary drying phase. The first feature is the linear region in the trajectory of the $C^{\prime }\left(100\;\mathrm{kHz}\right)$ time profile that results from a constant rate of reduction in the height of the ice cylinder, without any change in the shape of the sublimation interface. This region corresponds to a period of steady state and so identifies the time period during which heat, and mass transfer equations may be applied, rather than relying on a rule of thumb that the steady state period prevails over the first 25–30% loss of ice mass. The second feature is the deviation from linearity in the trajectory of the $C^{\prime }\left(100\;k\;\mathrm{Hz}\right)$ time profile which indicates the point when the shape of the sublimation interface begins to transform from an ice cylinder into an ice dome, due to the preferential drying of the ice at the interface with the glass wall. The third feature is that the $C^{\prime }\left(100\;\mathrm{kHz}\right)$ profile approaches a minimum as the ice cylinder continues to transform into an ice dome. At this lowest point in its trajectory the ice is no longer in contact with the side wall and thereafter the ice mass shrinks across the base of the vial. The fourth feature is that the de-coupling of the fringing field lines to the ground plane of the shelf (as the ice mass recedes across the base of the vial) means that the capacitance can now start to increase again, in what we have termed the recovery phase. The fifth feature is the end of the steep ascend of the $C^{\prime }\left(100\;\mathrm{kHz}\right)$ parameter which then marks the end of ice sublimation (as evidenced by the absence of ice in the photographic image of the vial). In the case of a full shelf of vials, an additional feature may be observed which is a more gradual ascent towards a plateau value when the whole system reaches thermal equilibrium.

## 5. Future Work

Our next studies will explore whether the characteristic profile from the high frequency capacitance of the TVIS vial is universal for solutions, as well as for ice. The cake is likely to impact (i.e., stabilize) the way the topography of the ice front develops, as so we might expect the same principal shape but with different durations for each phase; For example, the stabilization of the ice front might allow for a longer linear phase (from points A to B). The main difference, however, might be a result of the existence of the solids fraction that is left behind once the ice has been removed. It is known that this partially dry material liberates water from the desorption of that portion of water which did not crystalize as ice but instead hydrates the solutes within the solids fraction. The desorption of the adsorbed water requires energy and given that the vial heat transfer is poor then the energy is drawn to a large extent from the thermal mass of the vial and its contents. We have already seen, from this study, how a drift in the temperature of a vial can be observed after the sublimation end point is reached for pure water within that vial, as a consequence of the heat being removed by neighboring vials that are still in the active process of ice sublimation. We might also expect there to be a similar thermal effect from the desorption of water from the solids fraction within an individual vial and for this to contribute to an extension to the period D to E. 

The preliminary observations, that we have made to date, indicate similar behavior is indeed observed for simple sugar solutions. In subsequent studies, we shall be able to make some use of the photographic evidence from a front/edge vial; even though we might be able to see the shape of the ice front we can nevertheless detect the height of the ice cylinder in intimate contact with the inside perimeter of the vial; given that ice which intimate contact with the inside wall will take on a different appearance to that of the dried solids fraction and any ice fraction that has pulled away from the wall owing to preferential drying. We shall compare the photographic evidence for the rate of decrease in the height of the ice cylinder in contact with the side wall with the rate of decrease in the high frequency capacitance, and if both are linear with time then that will confirm that we can use the linear region (A to B) of the $C^{\prime }\left(100\;\mathrm{kHz}\right)$ time profile to establish that period when the ice interface is stable, in terms of its shape and hence surface area. 

The next point to qualify is the dip at point C, where the $C^{\prime }\left(100\;\mathrm{kHz}\right)$ parameter reaches its minimum value. Given that we expect this point to correspond to the condition when the ice mass has no contact with the sides of the glass wall, then we can again use the photographic evidence to establish whether this is indeed the case. 

The more challenging transition is to establish whether point D provides a universal indicator for the condition when all the ice has been removed from the frozen solution. Photographic images cannot see what is happening on the underside of the vial and so an alternative method is proposed, in which a series of TVIS vials is placed at various positions from the front (edge) to the core. The primary drying cycle is then interrupt once the $C^{\prime }\left(100\;\mathrm{kHz}\right)$ profile for the first two vials have transitioned beyond point D, but the others are yet to do so. On removing all vials at the end of the interrupted cycle then the base of the vial can be examined for evidence of melt-back of the ice fraction that had not yet fully sublimed. This experiment may need to be repeated a number of times, first with water (where melt-back will be obvious from a residue of liquid water) and then with a solution such as 5% mannitol, for example. A pure crystallizing excipient would be chosen, such as a mannitol after a process of annealing, as it can produce a robust cake that does not collapse when the temperature is increased. However, it will nevertheless show a ‘wet’ patch, and a local collapse of the cake, from the melt-back of any remaining ice fraction.

In industrial practice, the preference is for the determination of the sublimation end point is the use of a comparative pressure measurement (CPM) using a Pirani gauge and the capacitance manometer, given that this approach determines the batch end point rather than a single vial. However, when drying frozen solutions (as opposed to simply ice) the CPM approach will also detect water vapor generated from the unfrozen fraction as well as that from sublimation of ice. It is a characteristic of the time profile of the pressure measured by the Pirani gauge that it attains a steady state value when the chamber is saturated with moisture vapors but then as the sublimation of ice comes to an end, across the population of vials in the batch, the measured pressure decreases in a sigmoidal way. However, during this sigmoidal descent of the measured pressure, the Pirani gauge senses water vapors desorbing from the solids fraction and only comes to a new steady state value once an equilibrium has been achieved between the residual water in the solids fraction and the water vapor in the chamber. The two processes overlap which then obscures the true endpoint of the sublimation phase. 

The potential for TVIS to be able to differentiate between the ice sublimation end point and the ongoing desorption of moisture from the solids fraction, will be based largely on whether the sublimation of ice from frozen solutions results in a distinct inflection in the high frequency capacitance profile, from the steep, almost linear rise which then transitions abruptly to a more gradual rise once the individual vial sublimation end point has been reached. If this turns out to be the case, then TVIS might in future be used to provide a clue as to which characteristic of the CPM time profile can be taken as the true sublimation end point of the batch. 

## Figures and Tables

**Table 1:** Freeze-drying cycle for the TVIS vial containing 3 g of double distilled water.

Step	Start Temperature (°C)	End Temperature (°C)	Ramp Rate (°C/Min)	Duration (Min)	Cumulative Time (h)	Set Pressure (µbar)
Equilibrium phase	RT	20	-	15	0.3	-
Freezing	20	−40	0.6	100	1.9	-
Hold	−40	−40	-	120	3.9	-
Re-heating	−40	−10	0.5	60	4.9	-
Hold	−10	−10	-	60	5.9	-
Re-freezing	−10	−40	0.5	60	6.9	-
Hold	−40	−40	-	60	7.9	-
Re-heating	−40	−15	0.6	40	8.6	-
Hold	−15	−15	-	40	9.3	-
Primary drying	−15	−15	-	506	17.7	400

**Table 2:** Freeze-drying protocol for 3 g of double distilled water in 160 × 10R vials.

Step	Start Temperature (°C)	End Temperature (°C)	Ramp Rate (°C/Min)	Duration (Min)	Cumulative Time (h)	Set Pressure (µbar)
Equilibrium phase	RT	20	1	20	0.3	-
	20	20	-	10	0.5	
Freezing	20	−45	0.5	130	2.7	-
Hold	−45	−45	-	120	4.7	-
Re-heating	−45	−5	0.5	80	6.0	-
Hold	−5	−5	-	120	8.0	-
Primary drying	−5	−5	-	1928	40.1	400

## Data Availability

The data presented in this study are available at 10.21253/DMU.16910119.
